# High Concentration of C5a-Induced Mitochondria-Dependent Apoptosis in Murine Kidney Endothelial Cells

**DOI:** 10.3390/ijms20184465

**Published:** 2019-09-10

**Authors:** I-Jung Tsai, Wei-Chou Lin, Yao-Hsu Yang, Yu-Lin Tseng, Yen-Hung Lin, Chia-Hung Chou, Yong-Kwei Tsau

**Affiliations:** 1 Department of Pediatrics, National Taiwan University Hospital and National Taiwan University College of Medicine, Taipei 100, Taiwan; 2 Department of Pathology, National Taiwan University Hospital and National Taiwan University College of Medicine, Taipei 100, Taiwan; 3 Department of Internal Medicine, National Taiwan University Hospital and National Taiwan University College of Medicine, Taipei 100, Taiwan; 4 Department of Obstetrics and Gynecology, National Taiwan University Hospital and National Taiwan University College of Medicine, Taipei 100, Taiwan

**Keywords:** kidney endothelial cell, apoptosis, ROS regeneration, mitochondria, C5a

## Abstract

Patients with a relapse of idiopathic nephrotic syndrome have significantly increased levels of serum complement component 5a (C5a), and proteinuria has been noted in mice treated with C5a via changes in permeability of kidney endothelial cells (KECs) in established animal models. However, the apoptosis of KECs treated with high concentrations of C5a has also been observed. As mitochondrial damage is known to be important in cell apoptosis, the aim of this study was to examine the association between C5a-induced mouse KEC apoptosis and mitochondrial damage. Mouse KECs were isolated and treated with different concentrations of C5a. Cell viability assays showed that a high-concentration mouse recombinant protein C5a (rmC5a) treatment reduced mouse KEC growth. Cell cycle phase analysis, including apoptosis (sub-G1 phase) showed an increased percentage of the subG1 phase with a high-concentration rmC5a treatment. Cytochrome c and caspase 3/9 activities were significantly induced in the mouse KECs after a high-dose rmC5a (50 ng/mL) treatment, and this was rescued by pretreatment with the C5a receptor (C5aR) inhibitor (W-54011) and N-acetylcysteine (NAC). Reactive oxygen species (ROS) formation was detected in C5a-treated mouse KECs; however, W-54011 or NAC pretreatment inhibited high-dose rmC5a-induced ROS formation and also reduced cytochrome c release, apoptotic cell formation, and apoptotic DNA fragmentation. These factors determined the apoptosis of mouse KECs treated with high-dose C5a through C5aR and subsequently led to apoptosis via ROS regeneration and cytochrome c release. The results showed that high concentrations of C5a induced mouse KEC apoptosis via a C5aR/ROS/mitochondria-dependent pathway. These findings may shed light on the potential mechanism of glomerular sclerosis, a process in idiopathic nephrotic syndrome causing renal function impairment.

## 1. Introduction

Serum complement component 5a (C5a) is a small soluble peptide fragment generated from complement cascade activation that regulates many inflammatory pathways, such as the degranulation of mast cells [1], neutrophil chemotaxis [2], and T lymphocyte infiltration [3]. In non-myeloid cells, C5a has been reported to cause renal injuries, such as renal transplantation allograft survival and renal ischemia-reperfusion injury [4,5,6].

In the glomerular filtration barrier (GFB), the key mechanism regulating the barrier between podocytes and glomerular endothelial cells is unclear. Patients with early diabetes, pre-eclampsia and a rare hereditary nephrotic syndrome may present with proteinuria without any structural changes in podocytes [7,8,9]. Some studies have reported that podocytes produce vascular endothelial growth factor (VEGF) and may influence the function and survival of adjacent endothelial cells [10]. In our previous study, we demonstrated C5a-induced proteinuria in murine nephrotic syndrome via a Rho-associated kinase (ROCK) pathway in glomerular endothelial cells [11].

In general, endothelial cells play an important role in inflammatory processes such as atherosclerosis and sepsis [12,13,14,15]. C5a and C3a are known to be anaphylatoxins, to promote pro-inflammatory conditions, and to induce cell apoptosis. C5a is the more potent stimulant and can cause chemotaxis and produce oxidative substances by phagocytic cells [12,13,16]. In addition, C5a receptors (C5aR and CD88) exist in both myeloid cells (e.g., monocytes, macrophages and polymononuclear cells) and endothelial cells [17,18,19,20].

Recently, we identified that high concentrations of C5a may induce cellular apoptosis in kidney endothelial cells (KECs). This high concentration of C5a caused cellular apoptosis through C5a/C5aR in mouse KECs via cytochrome c and caspase 3/9 activities, which were dependent on reactive oxygen species (ROS) formation; as such, this apoptosis was mitochondrial-dependent.

## 2. Results

### 2.1. High-Dose C5a Treatment Reduced Mouse KEC Growth

**Figure 1:**
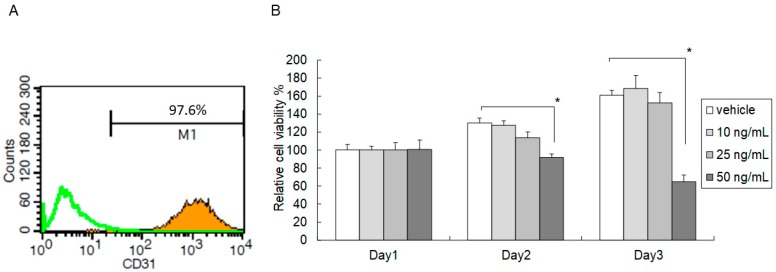
High-dose C5a treatment reduced mouse kidney endothelial cell (KEC) growth. (**A**) The ratio of CD31-positive cells were analyzed by flow cytometry using a FACScan and the Cell Quest software to quantify the CD31-positive cells. The white histograms were isotype controls, whereas the orange overlays were of CD31-positive cells. (**B**) Mouse KECs were treated with 0–50 ng/mL of Mouse recombinant protein C5a (rmC5a) for different periods of time. The cell viability was determined by an MTT assay. The data are presented as mean ± SD. * *p* < 0.05.

The growth inhibitory effect of C5a on mouse KECs was determined by an MTT assay under different concentrations of Mouse recombinant protein C5a (rmC5a) for three days. A high concentration (50 ng/mL) of rmC5a significantly reduced the relative viability of mouse KECs compared to the vehicle on day two and day three (Figure 1). These data suggested that a high dose of C5a reduced the growth of mouse KECs.

### 2.2. High-Dose C5a Treatment Induced Apoptosis of Mouse KECs

**Figure 2:**
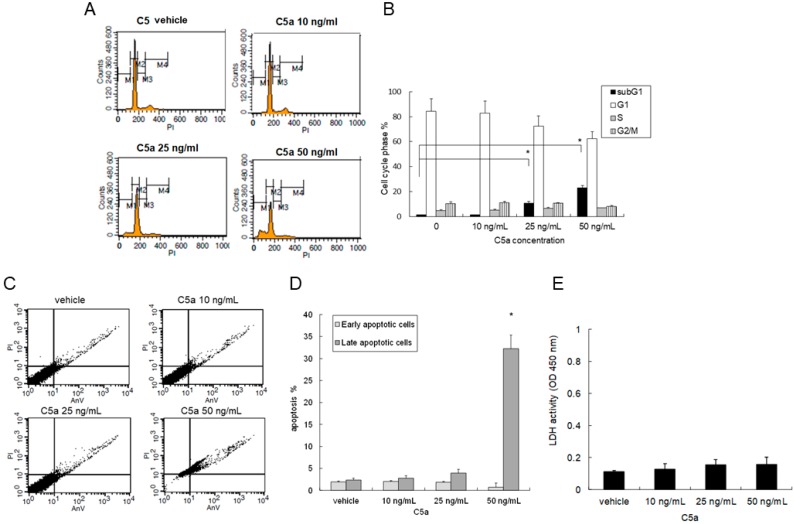
High-dose C5a treatment induced the apoptosis of mouse KECs. (**A**) Mouse KECs were treated with 0–50 ng/mL of rmC5a for 48 h. The cell cycle phases including apoptosis (sub-G1 phase) were analyzed by PI staining and flow cytometry. (**B**) The data are represented as mean ± SD. * *p* < 0.05. (**C**) Mouse KECs were treated with 0–50 ng/mL of rmC5a for 48 h. The early and late stage apoptotic cells were determined by staining with both PI and annexin V-FITC as well as flow cytometry. (**D**) The quantitative data are represented as mean ± SD. * *p* < 0.05. (**E**) The culture supernatant was collected from mouse KECs treated with 0–50 ng/mL of rmC5a for 48 h. Lactate dehydrogenase (LDH) activity of cell culture supernatant from each sample was measured by an LDH assay. The data are represented as the mean color intensity (OD 450 nm) ± SD of five independent analyses.

Mouse KECs were treated with rmC5a for 48 h, and the cell cycle phases including apoptosis (subG1 phase) were analyzed. The vehicle and 10 ng/mL of rmC5a did not change the cell cycle phases or induce an apoptosis of the mouse KECs. However, 25 ng/mL of rmC5a slightly but significantly induced a sub-G1 peak ratio, and 50 ng/mL of rmC5a markedly induced a sub-G1 peak ratio, which represented an apoptosis of the mouse KECs (Figure 2A,B). The early and late stage apoptotic cells were determined by staining both with propidium iodine (PI) and Annexin V-FITC, and 50 ng/mL of rmC5a induced a significant increase of apoptotic percentage in mouse KECs (Figure 2C,D). The lactate dehydrogenase (LDH) assay showed no difference between different concentrations of rmC5a. These results indicated that a high dose of C5a could induce mouse KEC apoptosis.

### 2.3. High-Dose C5a Treatment Induced Cytochrome c and Caspase 3/9 Activities through C5aR in Mouse KECs

**Figure 3:**
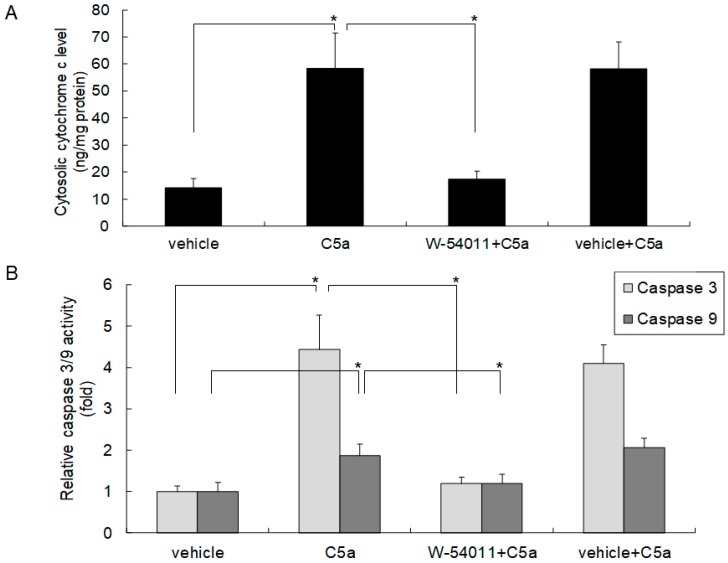
High-dose C5a treatment induced cytochrome c and caspase 3/9 activities through C5aR in mouse KECs. Mouse KECs were pretreated with the C5aR inhibitor (W-54011; 10 µg/mL) or vehicle (Dimethyl sulfoxide (DMSO); 0.1%) for 1 h prior to 50 ng/mL of rmC5a treatment. After 48 h, cytosolic protein was purified for (**A**) cytochrome c and (**B**) caspase 3/9 activities by ELISA. The data are represented as mean ± SD. * *p* < 0.05.

Apoptosis is associated with the activation of cytochrome c and caspase 3/9. To clarify the role of C5aR in apoptosis induced by C5a, mouse KECs were pretreated with the C5aR inhibitor W-54011 prior to C5a treatment. The results revealed that 50 ng/mL of rmC5a significantly induced cytochrome c release (Figure 3A) and caspase 3/9 activity (Figure 3B) in mouse KECs, whereas pretreatment with the C5aR inhibitor significantly rescued these induction effects (Figure 3). These results demonstrated that a high dose of C5a induced apoptosis through C5aR on mouse KECs.

### 2.4. High-Dose C5a Treatment Induced Oxidative Stress via NOXs-Dependent ROS Generation in Mouse KECs

**Figure 4:**
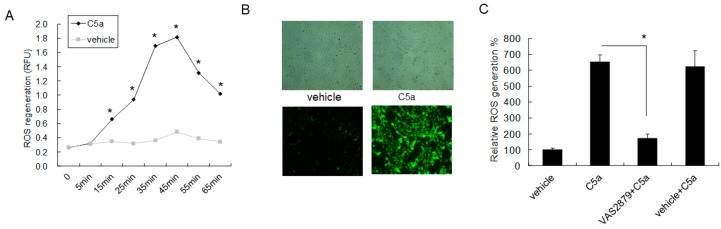
High-dose C5a treatment induced oxidative stress via NOXs-dependent reactive oxygen (ROS) generation in mouse KECs. (**A**) Mouse KECs were treated with vehicle or 50 ng/mL of rmC5a. The time sequence of ROS formation was detected by DCF-DA (Cellular Reactive Oxygen Species Detection Assay). (**B**) Representative florescence images of vehicle or 50 ng/mL of rmC5a-treated groups at 45 min were taken. (**C**) Mouse KECs were pretreated with pan NOXs inhibitor VAS2879 (10 µM) or vehicle (0.1% DMSO) for 30 min prior to 50 ng/mL of rmC5a treatment. After 45 min, ROS formation was determined by DCF-DA. The data are represented as mean ± SD. * *p* < 0.05.

The time sequence of ROS formation in rmC5a-treated mouse KECs is shown in Figure 4A. Based on the ROS formation curve, florescence images of vehicle or 50 ng/mL of rmC5a-treated groups at 45 min revealed ROS formation (Figure 4B). To clarify the role of NADPH oxidases (NOXs) in C5a-mediated ROS formation in KECs, pan NOXs inhibitor VAS2879 was used prior to C5a treatment in KECs. The results revealed VAS2879 significantly reduced C5a enhanced ROS generation in KECs (Figure 4C), which demonstrated that C5a triggered oxidative stress via NOXs-dependent ROS generation.

### 2.5. C5aR Inhibitors (W-54011) or NAC Rescued High-Dose C5a Treatment Induced ROS Formation and Apoptosis in Mouse KECs

**Figure 5:**
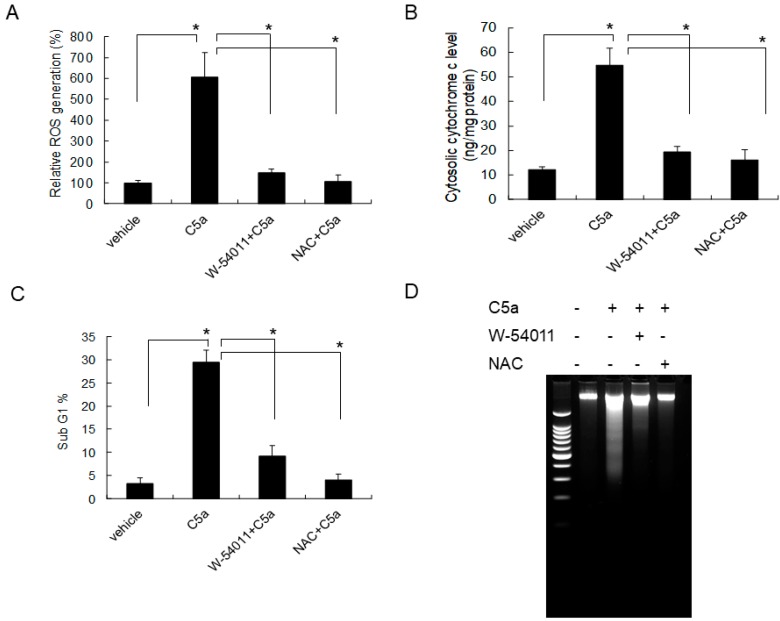
C5aR inhibitors (W-54011) or N-acetylcysteine (NAC) rescued high-dose C5a treatment-induced ROS formation and apoptosis in mouse KECs. (**A**) Mouse KECs were pretreated with the C5aR inhibitors (W-54011; 10 µg/mL) or NAC (1 mM) for 1 h prior to 50 ng/mL of rmC5a treatment. After 45 min, ROS formation was detected by DCF-DA assay. (**B**) Mouse KECs were pretreated with the C5aR inhibitors (W-54011; 10 µg/mL) or NAC (1 mM) for 1 h prior to 50 ng/mL of rmC5a treatment. After 48 h, cytosolic protein was purified to determine cytochrome c content using ELISA. (**C**) Mouse KECs were pretreated with the C5aR inhibitor (W-54011; 10 µg/mL) or NAC (1 mM) for 1 h prior to 50 ng/mL of rmC5a treatment. After 48 h, the apoptotic phase (subG1 phase) was analyzed by propidium iodide staining and flow cytometry. (**D**) Using the same treatment groups as described in (**C**), after 48 h, the total DNA of each group was purified to determine apoptotic DNA fragmentation using gel electrophoresis. The data are presented as mean ± SD. * *p* < 0.05.

To evaluate the role of C5aR in C5a-induced ROS formation, mouse KECs were pretreated with the C5aR inhibitor (W-54011) or N-acetylcysteine (NAC) prior to C5a treatment. A high dose of C5a induced ROS formation, and this effect was significantly inhibited by W-54011 or NAC (Figure 5A). Furthermore, the inhibition of C5a, which can induce ROS formation through the C5aR inhibitors (W-54011) or NAC, also reduced cytochrome c release (Figure 5B), apoptotic cell formation (Figure 5C), and attenuated apoptotic DNA fragmentation (Figure 5D). Taken together, these results indicated that a high dose of C5a may damage mouse KECs through C5aR/ROS/mitochondria-dependent apoptosis.

## 3. Discussion

C5a is cleaved from complement component C5 by C5-convertase in the complement cascade, and it is an active peptide in anaphylactic reactions and inflammatory processes. C5a stimulates mast cell degranulation, stimulates the release of tumor necrosis factor-α (TNF-α) and histamine, and recruits phagocytes to sites of infection and inflammation by increasing adhesion molecule expression on the surface of endothelial cells [21,22]. C5a also increases vascular permeability in some pathological stimuli, such as allograft rejection after transplantation and asthma [4,23,24] The serum level of C5a has been discussed in several studies. In a study by Lechner et al., the C5a level was 8.34 + 2.05 (ng/mL) in the control group [25]. Under normal conditions, the plasma C5a level is quite low [26,27] because of the rapid clearance of anaphylatoxin [28]. In our previous study, the nephrotic syndrome patients with relapse showed a higher serum C5a level (44.30 + 32.95 ng/mL) compared to remission status (22.31 + 14.62 ng/mL) [11]. In murine cortical tubular cells, after treatment with C5a (25 nM), transforming growth factor-β (TGF-β) was elevated which has been shown to lead to renal fibrosis and renal scar formation [29].

In the GFB, the key mechanism regulating the barrier between podocytes and glomerular endothelial cells is still under debate. Due to the presence of large fenestrations of glomerular endothelial cells, the endothelium has not traditionally been considered to be a major contributor to GFB function, and massive proteinuria has been noted when certain proteins on podocytes are defective or absent, such as in congenital nephrotic syndrome [30,31]. However, the role of glomerular endothelial cells has recently been reconsidered. In a diabetic mice model, endothelial nitric oxide synthetase deficiency was shown to cause heavy proteinuria and structural changes of podocytes in a similar manner to podocyte changes in minimal change disease [32]. In a study of the effect of angiopoietin-2 on podocytes, the overexpression of angiopoietin-2 induced the apoptosis of glomerular endothelial cells and the presence of albuminuria; however, the structure of podocytes remained intact [33].

C5a is a potent proinflammatory molecule [34] which binds to C5aR (CD88), a classical G protein-coupled receptor (GPCR), and elicits the signaling pathways of proinflammatory responses [35]. C5aR is expressed in different non-myeloid cells, such as human umbilical vascular endothelial cells (HUVEC), murine dermal, liver, pulmonary and renal proximal tubules [20,36,37,38,39]. We previously demonstrated that C5aR was expressed in glomerular endothelial cells but not in podocytes, indicating that C5a may cause proteinuria on the primary target of renal endothelial cells [11].

Previous studies have shown that C5a may induce lymphocyte apoptosis which presents as immunosuppression in severe sepsis, and in some critical conditions, the loss of regulation of C5a may cause plasma concentrations in excess of 100 nmol/L [13,27,40]. In sepsis, high concentrations of proinflammatory mediators, such as C5a, may be associated with apoptotic cell death. In a HUVEC study, C5a enhanced the gene expressions of chemokines (interleukin-1/interleukin-6), cell adherence molecules (intercellular adhesion molecule-1 (ICAM-1)/vascular cell adhesion protein 1 (VCAM-1)/E-selectin), growth factors VEGF/platelet-derived growth factor (PDGF)), and pro-apoptotic molecules (caspase 3, caspase 8, cellular FLICE inhibitory protein (cFLIP)) [41]. In addition, C5a has been shown to affect ROS production in neutrophils, and C5a-induced apoptosis has been shown to be dependent on phosphoinositide signaling [42,43], which then reduces endothelial integrity [44]. In recent studies of lupus, C5a/C5aR1 signaling was shown to induce the apoptosis of brain endothelial cells, and this effect was inhibited by C5a/C5aR1 antagonists [45,46]. Furthermore, in a study by Drummond et al., it was shown that NOXs are possible mediators in C5a-triggering oxidative stress [47].

Apoptosis is a procedure of active cell death—an energy-requiring process—which regulates the number of cells [48,49]. In addition, apoptosis also regulates immune and inflammatory cells, the number of fibroblasts, and vascular homeostasis [50]. In regard to kidney cells, the apoptosis of renal parenchymal cells is related to cell damage, which causes a decrease in the number of kidney epithelial cells [51,52,53], leading to acute or chronic kidney injury. During apoptosis, the mitochondria is the most important factor in the intrinsic pathway, which produces pre-apoptotic factors, including cytochrome c, apoptosis-inducing factor (AIF) and Smac/DIABLO [54,55], causing proteolysis and cell death. In kidney cells, cascade-dependent apoptosis and cell death can be caused by nephrotoxic agents in glomerular and tubular epithelium through mitochondrial injury and caspase cascade activation [50]. For example, in cyclosporin A-induced nephrotoxicity, Bax aggregation and translocation to the mitochondria in renal tubular cells may change the permeability of outer mitochondrial membranes to release cytochrome c and Smac/DIABLO and then activate caspase 3/9, which causes cell apoptosis and death [56]. However, the apoptosis pathways in KECs are still unclear.

Our results demonstrated the significant apoptosis of mouse KECs treated with high concentrations of rmC5a, which decreased cell viability and increased the sub-G1 phase in cell cycle phase analysis. In addition, our data also showed that rmC5a signaling was done through C5aR on mouse KECs, and that this induced cytochrome c and caspase 3/9 activities which were dependent on ROS regeneration; therefore, this was mitochondrial-dependent apoptosis. In advance, C5a-triggered oxidative stress may be through NOXs-dependent ROS generation. Of note, this study is the first to show that a high dose of C5a may damage mouse KECs through C5aR, thereby causing mitochondrial-dependent apoptosis. This is in line with the beneficial effects of the C5a/C5a receptor blockade in treating experimental sepsis. Our findings not only shed light on the potential mechanism of glomerular sclerosis in idiopathic nephrotic syndrome, a process of renal impairment, but also a possible direction for novel therapeutic approaches in complement-mediated KEC apoptosis.

## 4. Materials and Methods

### 4.1. Mouse Kidney Endothelial Cell (KEC) Preparation

The kidney glomeruli of 20 mice aged between 6 and 8 weeks were isolated and minced in sterile conditions by stepwise sieving with stainless steel sieves of different pole sizes (150, 90, 75, 53, and 32 µm). The isolated glomeruli were then washed in 50 mL of Dulbecco’s modified Eagle’s medium (DMEM) (Invitrogen) followed by a treatment at 37 °C for 1.5 h with 2.5 mL of 0.1% type II collagenase in DMEM. Five ml of Hanks’ balanced salt solution containing 10% fetal calf serum (Invitrogen) was then used to wash the cell suspension. The endothelial cells from mouse kidney tissue were isolated by immunomagnetic purification with anti-CD31–coated Dynabeads^®^. The isolated endothelial cells were resuspended in 1 mL of a cold M199 medium containing 0.3 mg/mL of magnetic tosyl-activated M450 Dynabeads with mouse anti-mouse CD31, and they were then incubated at room temperature for 30 min with gentle agitation. An MPC-1 magnet (Dynal, Oslo, Norway) was used to isolate endothelial cells bound to the magnetic beads from the unbound non-endothelial cells. The magnetic bead-bound cells were resuspended and cultured in an endothelial cell growth medium (Cell Applications, San Diego, CA, USA). The ratio of CD31-positive cells was analyzed by quantifying the CD31-positive cells (stained by PECAM-1 which also referred to as CD31 Antibody; sc-376764 FITC) by flow cytometry using a FACS scan and the Cell Quest software (Becton Dickinson Immunocytometry Systems, San Jose, CA, USA). All animal experiments were performed according to the protocols approved by the Institutional Animal Care and Use Committee (IACUC) of National Taiwan University College of Medicine (No. 20160138), and the care and use of the animals was in accordance with the guidelines of National Institutes of Health (NIH).

### 4.2. Recombinant Protein and Chemical Reagents

Mouse recombinant protein C5a (rmC5a) was purchased from R&D Systems. The C5aR inhibitor W-54011, VAS2870, DMSO, DCF-DA and NAC were purchased from Sigma-Aldrich (St. Louis, MO, USA).

### 4.3. Cell Viability Determination

Cells were cultured in 96-well plates at 2 × 1000 cells/well with individual experimental conditions. The growth inhibitory effects were measured. At the indicated time periods, we added 30 μL of a 5 mg/mL MTT solution (Sigma-Aldrich, St. Louis, MO, USA) to each well and then incubated the cells at 37 °C for 4 h. The cell culture supernatant was then aspirated. The developed formazan crystals were then dissolved in 200 μL of DMSO (Sigma-Aldrich, St. Louis, MO, USA) for 10 min, and an ELISA reader at 540 nm was used to analyze the absorbance. The percentage of viability in control cells was presented as absorbance values. All values were presented as mean ± standard deviation (SD) of at least three independent experiments.

### 4.4. Cell Cycle and Apoptosis Analysis Determined by Flow Cytometry

Cells were cultured in a six-well plate (50,000 cells/well) and incubated overnight. After experimental treatment, the attached cells were collected and fixed with methanol at −20 °C for at least 30 min and then stained with 10 mg/mL of propidium iodide and 100 mg/mL of RNase A for 30 min in the dark. Flow cytometric analysis (FACSCalibur; Becton Dickinson, San Jose, CA, USA and Cell Quest software (Becton Dickinson) were used to determine cell cycle distribution. For apoptosis analysis, KECs were resuspended in 100 µL of an Annexin V (AV) binding buffer (10 mM HEPES (pH 7.4), 140 mM NaCl, and 2.5 mM CaCl_2_) and then added to fluorescein isothiocyanate-conjugated AV (5 µL; Invitrogen Corporation, Carlsbad, CA, USA) and PI (1 µL, 1 mg/mL; Invitrogen, Carlsbad, CA, USA). After incubation for 20 min, 400 uL of the annexin V binding buffer was added, and the cells were analyzed by flow cytometry as described above. All values were presented as mean + standard deviation (SD) of at least three independent experiments.

### 4.5. Lactate Dehydrogenase (LDH) Assay

The cell culture supernatant of KECs was processed for measuring LDH activities by using an Abcam’s LDH assay kit (Colorimetric: ab102526) according to the manufacturer’s protocols. 50 µL of culture supernatant from each sample was collected and combined with 50 µL of Reaction Mix (contain the LDH Assay Buffer and the LDH Substrate Mix) at 37 °C protected from light. After 30 min, the LDH activity was measured at 450 nm using a microplate reader (Bio-Rad Laboratories, Hercules, CA, USA).

### 4.6. Cytochrome c Release and Caspase 3/9 Activity Determination

Cells were cultured in a six-well plate (50,000 cells/well) and incubated overnight. After experimental treatment, cytosolic protein was purified to determine cytochrome c and caspase 3/9 activities using a Rat/Mouse Cytochrome c Quantikine ELISA Kit (R&D Systems, Minneapolis, MN, USA), Caspase-3/CPP32 Colorimetric Assay Kit (R&D Systems) and Caspase-9 Colorimetric Assay Kit (R&D Systems), respectively, according to the manufacturer’s instructions. The cytosolic proteins of KECs were isolated by Abcam′s Cell Fractionation Kit (Standard Cell Fractionation Kit – Standard; ab109719). KECs cells were suspended in buffer A, and then the cell suspension was diluted with an equal volume of buffer B in 1.5 mL microcentrifuge tubes containing protease inhibitors, and the tube was incubated with constant mixing for 7 min on a rotator at room temperature. Then, samples were centrifuged at 5000× *g* for 1 min at 4 °C. All supernatants were carefully removed and transferred to a new set of tubes. The supernatant fractions were re-centrifuged at 10,000× *g* for 1 min. Again, all of the supernatants were carefully removed and transferred to a new set of tubes. The concentration of cytosolic proteins was quantified using a Bio-Rad protein assay reagent (Bio-Rad Laboratories, Hercules, CA, USA).

### 4.7. ROS Formation Determination by DCF-DA

Changes in the ROS content in cells were determined using the fluorescent dye DCF-DA. KECs were placed in a six-well plate (50,000 cells/well) 48 h before the experiment. The cells were then cultured in a serum-free medium for 6 h followed by incubation with 10 µmol/L of DCF-DA (Sigma, Saint Louis, MO, USA) for 30 min. DCF-DA fluorescent emission was measured using a Beckman Coulter DTX 880 Multimode Detector at 530 nm, with excitation at a wavelength of 488 nm.

### 4.8. Visualization of DNA Fragmentation with Gel Electrophoresis

DNA was extracted from cells by the phenol–chloroform method. Electrophoresis was performed on 2% agarose gels in a Tris/acetate acid/EDTA buffer. The gels were stained with ethidium bromide to show the DNA fragments and photographed with a digital camera.

### 4.9. Statistical Analysis

All data were expressed as mean ± SD. In cell studies, a two-tailed *t*-test was used to evaluate statistically significant differences between two groups. A *p* value < 0.05 was considered to indicate a statistically significant difference.
